# *MMP-3* gene polymorphisms are associated with increased risk of osteoarthritis in Chinese men

**DOI:** 10.18632/oncotarget.18493

**Published:** 2017-06-15

**Authors:** Wen Guo, Pengcheng Xu, Tianbo Jin, Jihong Wang, Dongsheng Fan, Zengtao Hao, Yuntao Ji, Shangfei Jing, Chaoqian Han, Jieli Du, Dong Jiang, Shuzheng Wen, Jianzhong Wang

**Affiliations:** ^1^ Inner Mongolia Medical University, Hohhot, Inner Mongolia, China; ^2^ National Engineering Research Center for Miniaturized Detection Systems, School of Life Sciences, Northwest University, Xi’an, Shaanxi, China; ^3^ Xi’an Tiangen Precision Medical Institute, Xi’an, Shaanxi, China; ^4^ Department of Hand Surgery, Hebei Province Cangzhou Hospital of Integrated Traditional and Western Medicine, Cangzhou, Hebei, China; ^5^ Department of Trauma, Second Affiliated Hospital, Inner Mongolia Medical University, Hohhot, Inner Mongolia Autonomous Region, China; ^6^ Department of Hand Surgery, Hebei province Cangzhou Hospital of integrated Traditional and Western Medicine, Cangzhou, Hebei, China; ^7^ Cangzhou People’s Hospitial, Cangzhou, Hebei, China

**Keywords:** association, osteoarthritis, MMP-3, single nucleotide polymorphism

## Abstract

Osteoarthritis (OA) is the most common late-onset degenerative joint disease., It is characterized by progressive degradation of articular cartilage. We investigated the association between OA occurrence and single nucleotide polymorphisms (SNPs) in the *matrix metalloproteinase-3* (*MMP-3*) gene involved in the breakdown of extra-cellular matrix proteins. The study included 100 male OA patients and 197 healthy men from the north area of China. Eight *MMP-3* SNPs were genotyped. Odds ratios (ORs) with 95% confidence intervals (95%CIs) and multivariate logistic regression analysis were used to assess the association. Multivariate logistic regression analysis was used to identify SNPs that correlated with OA susceptibility. We found that rs639752 (dominant, OR = 2.03, 95% CI: 1.03-4.01, *P* = 0.038; over-dominant, OR = 2.00, 95% CI: 1.03-3.88, *P* = 0.037); rs520540 (dominant, OR = 2.03, 95% CI: 1.03-4.01, *P* = 0.038; over-dominant, OR = 2.00, 95% CI: 1.03-3.88, *P* = 0.037); rs602128 (dominant, OR = 2.03, 95% CI: 1.03-4.01, *P* = 0.038; over-dominant, OR = 2.01, 95% CI: 1.03-3.89, *P* = 0.037); and rs679620 (dominant, OR = 2.03, 95% CI: 1.03-4.01, *P* = 0.038; over-dominant, OR = 2.04, 95% CI: 1.05-3.96, *P* = 0.033) were associated with the increased risk of OA. Our results suggest that these SNPs may contribute to OA development, and could serve as molecular markers of OA susceptibility.

## INTRODUCTION

Bone and joint diseases are the most common causes of severe chronic pain and physical disability among elderly people, and affect the health of millions of people worldwide [1]. Osteoarthritis (OA), the most common late-onset degenerative joint disease, primarily affects the knees, hips, hands, and spine [2]. OA starts at weight-bearing areas, but progresses to peripheral regions [3]. The prominent features of OA include progressive degradation of articular cartilage, accompanied with joint space narrowing, subchondral bone sclerosis, and osteophyte formation at the joint margin, resulting in chronic joint pain and restricted motion [4]. Both genetic and non-genetic factors contribute to OA initiation and progression. The non-genetic factors include obesity, history of arthrosis injury, occupational activities, sex hormones and structural changes, meniscectomy, gender, and age [5]. Genetic factors may account for 40-60% [6]. A number of genes involved in development of knee osteoarthritis have been identified, such as *GDF5* [7], *ASPN* [8], *FRZB* [9], and *COL2*A*1* [10]*.* However, the genetic etiology of OA is still not completely clear [11]. A better understanding of the genetic factors underlying the development of OA is neededso that to identify high-risk individuals for targeted screening and prevention.

Matrix metalloproteinase-3 (MMP-3), also known as stromelysin-1, is a member of the MMPs family, which consists of 28 zinc-dependent endopeptidases [12]. MMP-3 is produced by various cell types including fibroblasts, smooth muscle cells, chondrocytes, synoviocytes, and macrophages [13-15]. under both are the pathology of (, also known as , expressed by [15]. It is involvedins’ andIt is involved in the breakdown of extra-cellular matrix proteins during normal physiological processes, such as embryonic development, reproduction, and tissue remodeling, as well as in disease processes, such as arthritis [16, 17].

A previous study has indicated that the -1612 5A/6A polymorphism genotypes of *MMP-3* gene promoter do not play a significant role in the OA development in Thai population [11]. rmIn this case-control study, we have analyzed whether eight single nucleotide polymorphisms (SNPs) identified in the *MMP-3* gene are associated with OA susceptibility in men from the north area of China.

## RESULTS

### Participant characteristics

A total of 100 male OA patients and 197 healthy men were enrolled in our study. The mean age of the participants was 51.18±7.849 years in the control group and 63.35±5.786 years in the OA case group. Since there was a significant difference in age between OA patients and control subjects (*p* < 0.01), we have adjusted for age in the subsequent data analysis.

### Association between *MMP-3* gene polymorphisms and OA risk

Detailed information about the selected SNPs is shown in Table 1. The minor allele of each SNP was compared with the wild-type allele. All tested SNPs were in Hardy-Weinberg equilibrium (HWE) in the control group (*P*>0.05). Comparing the differences in frequency distributions of alleles between cases and controls by χ^2^ tests, we found no correlation between the loci and the risk for OA under the allele model.

**Table 1:** Basic information of candidate SNPs in this study.

	Nucleotide		Allele	MAF		HW	Allele model		
SNPs	Position	Role	A/B	case	control	p-value	OR	95%CI	*P*-value
rs639752	102707339	Intron	C/A	0.395	0.360	1.000	1.159	0.816-1.645	0.410
rs650108	102708787	Intron	G/A	0.480	0.449	0.474	1.132	0.805-1.592	0.477
rs520540	102709425	Coding exon	A/G	0.395	0.360	1.000	1.159	0.816-1.645	0.410
rs646910	102709522	Intron (boundary)	A/T	0.075	0.089	1.000	0.832	0.443-1.562	0.566
rs602128	102713465	Coding exon	A/G	0.395	0.359	1.000	1.166	0.821-1.656	0.391
rs679620	102713620	Coding exon	T/C	0.395	0.363	1.000	1.146	0.808-1.626	0.445
rs678815	102713777	Intron	G/C	0.357	0.360	1.000	0.986	0.676-1.437	0.941
rs522616	102715048	Promoter	C/T	0.350	0.352	0.756	0.991	0.694-1.416	0.961

SNPs: Single nucleotide polymorphisms; MAF: Minor allele frequency; HWE: Hardy-Weinberg equilibrium; OR: Odds ratio; CI: Confidence interval. A: Minor alleles. B: Major alleles.

After adjusting for age, further model association analyses were performed by unconditional logistic regression analysis (Table 2). We found that rs639752 was associated with an increased OA risk by dominant (e genotype “CA-CC”, OR = 2.03, 95% CI: 1.03-4.01, *P* = 0.038) and over-dominant model analyses (“CA”, OR = 2.00, 95% CI: 1.03-3.88, *P* = 0.037). In the dominant and over-dominant models, rs520540 was associated with increased OA risk (dominant, “AG-AA”, OR = 2.03, 95% CI: 1.03-4.01, *P* = 0.038; over-dominant, “AG”, OR = 2.00, 95% CI: 1.03-3.88, *P* = 0.037). Rs602128 increased the risk of OA in dominant (“GA-AA”, OR = 2.03, 95% CI: 1.03-4.01, *P* = 0.038) and over-dominant model analyses (“GA”, OR = 2.01, 95% CI: 1.03-3.89, *P* = 0.037). Rs679620 was also associated with increased OA risk in dominant (“TC-TT”, OR = 2.03, 95% CI: 1.03-4.01, *P* = 0.038) and over-dominant model analyses (“TC”, OR = 2.04, 95% CI: 1.05-3.96, *P* = 0.033). There was no association between *MMP-3* loci and OA susceptibility using Bonferroni correction. In addition, no association was observed between SNP haplotypes and OA risk using the Wald test and unconditional multivariate regression analysis (Table 3).

**Table 2:** Single loci association with OA (adjusted by age).

SNPs	Model	Genotype	Controls(n%)	Cases(n%)	OR (95% CI)	*P*-value	AIC	BIC
rs639752	Codominant	A/A	80 (40.6%)	34 (34%)	1[Ref]			
		C/A	92 (46.7%)	53 (53%)	2.23 (1.08-4.59)	0.085	236.4	251.2
		C/C	25 (12.7%)	13 (13%)	1.50 (0.54-4.13)			
	Dominant	A/A	80 (40.6%)	34 (34%)	1[Ref]			
		C/A-C/C	117 (59.4%)	66 (66%)	2.03 (1.03-4.01)	0.038*	235	246.1
	Recessive	A/A-C/A	172 (87.3%)	87 (87%)	1[Ref]			
		C/C	25 (12.7%)	13 (13%)	0.96 (0.38-2.43)	0.940	239.3	250.4
	Over-dominant	A/A-C/C	105 (53.3%)	47 (47%)	1[Ref]			
		C/A	92 (46.7%)	53 (53%)	2.00 (1.03-3.88)	0.037*	235	246.1
	Log-additive	---	---	---	1.41 (0.88-2.26)	0.150	237.3	248.4
rs520540	Codominant	G/G	80 (40.6%)	34 (34%)	1[Ref]			
		A/G	92 (46.7%)	53 (53%)	2.23 (1.08-4.59)	0.085	236.4	251.2
		A/A	25 (12.7%)	13 (13%)	1.50 (0.54-4.13)			
	Dominant	G/G	80 (40.6%)	34 (34%)	1[Ref]			
		A/G-A/A	117 (59.4%)	66 (66%)	2.03 (1.03-4.01)	0.038*	235	246.1
	Recessive	G/G-A/G	172 (87.3%)	87 (87%)	1[Ref]			
		A/A	25 (12.7%)	13 (13%)	0.96 (0.38-2.43)	0.940	239.3	250.4
	Over-dominant	G/G-A/A	105 (53.3%)	47 (47%)	1[Ref]			
		A/G	92 (46.7%)	53 (53%)	2.00 (1.03-3.88)	0.037*	235	246.1
	Log-additive	---	---	---	1.41 (0.88-2.26)	0.150	237.3	248.4
rs602128	Codominant	G/G	80 (41%)	34 (34%)	1[Ref]			
		G/A	90 (46.1%)	53 (53%)	2.24 (1.09-4.60)	0.084	236.3	251
		A/A	25 (12.8%)	13 (13%)	1.49 (0.54-4.12)			
	Dominant	G/G	80 (41%)	34 (34%)	1[Ref]			
		G/A-A/A	115 (59%)	66 (66%)	2.03 (1.03-4.01)	0.038*	234.9	246
	Recessive	G/G-G/A	170 (87.2%)	87 (87%)	1[Ref]			
		A/A	25 (12.8%)	13 (13%)	0.96 (0.38-2.42)	0.940	239.2	250.3
	Over-dominant	G/G-A/A	105 (53.9%)	47 (47%)	1[Ref]			
		G/A	90 (46.1%)	53 (53%)	2.01 (1.03-3.89)	0.037*	234.9	245.9
	Log-additive	---	---	---	1.41 (0.88-2.26)	0.150	237.2	248.3
rs679620	Codominant	C/C	80 (40.6%)	34 (34%)	1[Ref]			
		T/C	91 (46.2%)	53 (53%)	2.26 (1.10-4.66)	0.078	236.2	251
		T/T	26 (13.2%)	13 (13%)	1.45 (0.53-3.98)			
	Dominant	C/C	80 (40.6%)	34 (34%)	1[Ref]			
		T/C-T/T	117 (59.4%)	66 (66%)	2.03 (1.03-4.01)	0.038*	235	246.1
	Recessive	C/C-T/C	171 (86.8%)	87 (87%)	1[Ref]			
		T/T	26 (13.2%)	13 (13%)	0.93 (0.37-2.32)	0.880	239.3	250.4
	Over-dominant	C/C-T/T	106 (53.8%)	47 (47%)	1[Ref]			
		T/C	91 (46.2%)	53 (53%)	2.04 (1.05-3.96)	0.033*	234.8	245.8
	Log-additive	---	---	---	1.39 (0.87-2.22)	0.170	237.4	248.5

SNPs: Single nucleotide polymorphisms; OR: Odds ratio. CI: Confidence interval.

*P* -value was calculated by Wald test. *p-value < 0.05 indicates statistically significant.

**Table 3:** Haplotype frequencies and their association with OA risk in case and control subjects.

		Frequency		Without adjustment		With adjustment	
SNPs	Haplotype	case	control	OR(95% CI)	*P*^a^	OR(95% CI)	*P*^b^
rs639752/rs650108/rs520540	CGATATGT	0.395	0.360	1[Ref]	---	1[Ref]	---
/rs646910/rs602128/rs679620	AAGTGCCC	0.345	0.352	0.90 (0.60 - 1.35)	0.61	0.77 (0.46 - 1.31)	0.33
/rs678815/rs522616	AAGTGCCT	0.175	0.199	0.81 (0.49 - 1.34)	0.41	0.72 (0.37 - 1.43)	0.35
	AGGAGCCT	0.075	0.089	0.78 (0.40 - 1.53)	0.47	0.47 (0.19 - 1.16)	0.10

OR: odd ratio; CI: confidence interval.

*P*-value <0.05 indicates statistical significance.

*P* ^*a*^ values were calculated from two-side Chi-square tests.

*P* ^*b*^ values were calculated by unconditional logistic regression adjusted for age.

## DISCUSSION

In this study, we have investigated the association between SNPs in the *MMP-3* gene and OA susceptibility in men from the north area of China. We have found that four SNPs (rs639752, rs520540, rs602128, and rs679620) are associated with the increased risk of OA in the dominant and over-dominant model.

Previous studies have indicated correlation between *MMP-3* polymorphisms and different diseases [18-24]. Menezes-Silva et al [18] have reported that *MMP-3* rs639752 and rs679620 genotypes are associated with the development of periapical lesions. In addition, Letra et al [19] have suggested that *MMP-3* is associated with chronic periodontitis in the US (rs679620) and Brazilian (rs639752) population. Since SNP rs679620 is a missense mutation that alters the *MMP-3* function [20], this mutation may affect bone remodeling, wound healing, as well as inflammatory responses. Indeed, h *MMP-3* rs679620 has been associated with the increased risk of tendon pathologyat. In addition, the *MMP-3* rs679620 variant has been shown to interact with the *COL5A1* rs12722 variant to modify the risk of tendinopathy [23]. Clearly, the mechanisms of how the *MMP-3* gene contributes to osteoarthritis are complex, and need to be clarified.

To the best of our knowledge, this is the first study indicating an association between *MMP-3* polymorphism and OA susceptibility. Although this study had a sufficient statistical power, there are some limitations. First, the sample size was not sufficient for association studies (100 cases and 197controls). Therefore, our findings must be confirmed in larger datasets as well as in a meta-analysis. Additionally, our study included only Han Chinese men. Epidemiological studies have demonstrated significantly increased occurrence of primary osteoarthritis in women compared to men [25], suggesting that the increased OA incidence might be caused by decreased estrogen levels in elderly women. Since we wanted to exclude the influence of estrogen, we have included only men in this study. Future studies should address the sex differences in the genetics of OA, as well as the effect of obesity, history of arthrosis injury, occupational activities, sex hormones, and structural changes. The fact that we have found no statistically significant association between *MMP-3* SNPs and OA susceptibility using Bonferroni correction may be caused by the relatively small sample size, the selection criteria for *MMP-3* SNPs (MAF > 5%), and the weakness of Bonferroni correction itself. True differences may have been deemed non-significant due to the type II errors.

In conclusion, our study has revealed a significant association between four polymorphisms (rs639752, rs520540, rs602128, and rs679620) in the *MMP-3* gene and increased risk of OA in men from the north area of China. Our results suggest that these SNPs may contribute to the OA development, and serve as molecular markers of OA susceptibility.

## MATERIALS AND METHODS

### Study participants

From January 2014 to July 2016, we recruited 100 male OA patients and 197 healthy men in this study. The patients were treated at the First Department of Trauma & Second Hand and Foot Surgery, Second Affiliated Hospital, Inner Mongolia Medical University, China. All demographic and clinical data including residential region, age, ethnicity, and education status were collected through a face-to-face questionnaire and a review of medical records. Patients recently diagnosed with primary OA were included in the study. The diagnosis criteria of OA were based on the American College of Rheumatology, and included primary OA with symptoms and radiographic signs of OA according to the Kellgren-Lawrence grading system [26]. The controls were recruited from Physical Examination Center in the first Affiliated Hospital, Inner Mongolia Medical University, China, and had no personal or family history of OA. Participants were excluded on the basis of having arthropathy due to gout, pseudogout, rheumatoid arthritis (RA), systemic lupus erythematosus, psoriasis, hemochromatosis, previous knee injury, or previous joint infection. In addition, patients with any systemic inflammatory or autoimmune disorder, or any type of malignant or chronic illness were not included in this study.

This study was performed in accordance with the Chinese Department of Health and Human Services regulations for the protection of human research subjects. Informed consents were obtained from all participants and the study protocols were approved by the Institutional Review Board of Inner Mongolia Medical University.

### SNP selection and genotyping

Validated SNPs, associated with other diseases in previous studies, were selected with a minor allele frequency (MAF) >5 % in the HapMap Asian population [13, 18, 21, 27-29]. After recruitment, venous blood samples (5 mL) were collected from each patient during a laboratory examination. DNA was extracted from whole blood samples using the Gold Mag-Mini Whole Blood Genomic DNA Purification Kit (GoldMag Ltd., Xi’an, China) and stored at -80^o^C after centrifugation. The Sequenom MassARRAY Assay Design 3.0 software (Sequenom, Inc, San Diego, CA, USA) was used to design the multiplexed SNP Mass EXTEND assay. Genotyping was performed using a Sequenom MassARRAY RS1000 (Sequenom, Inc.) according to the manufacturer’s protocol [30]. The SequenomTyper 4.0 Software™ (Sequenom, Inc.) was used to analyze the data [31]. The primers corresponding to each SNP are shown in Table 4. The following eight SNPs in *MMP-3* gene were selected: rs639752, rs650108, rs520540, rs646910, rs602128, rs679620, rs678815, and rs522616. The SNP data are shown in Table 1.

**Table 4:** Primers used for this study.

SNP_ID	1st-PCRP	2nd-PCRP	UEP_SEQ
rs639752	ACGTTGGATGCAGATAAATTCTCCACTTGC	ACGTTGGATGGGCTGCAATGCGGGAAAAG	tGGGAAGAAAGAAATAGGTGAT
rs650108	ACGTTGGATGGTCACTGTCTCATTGTGTGT	ACGTTGGATGTCAGGTAGAGGTGACAAGTG	tAAGTGGGTGAGGTTAGA
rs520540	ACGTTGGATGGCGAAAGGGCTTAACTGTTAT	ACGTTGGATGCCAGCTCGTACCTCATTTCC	CTCGTACCTCATTTCCTCTGAT
rs646910	ACGTTGGATGCCACTGTAAGCTGGTGACTA	ACGTTGGATGGTTAAGCCCTTTCGCTTTAG	CGCTTTAGAAATACACTTTAGCATCT
rs602128	ACGTTGGATGCTTCGGGATGCCAGGAAA	ACGTTGGATGAAGCTGGACTCCGACACTCT	CAGGTGTGGAGTTCCTGA
rs679620	ACGTTGGATGAACAGGACCACTGTCCTTTC	ACGTTGGATGAGAAATATCTAGAAAACTAC	tcTCTAGAAAACTACTACGACCTC
rs678815	ACGTTGGATGAATGCAACGTAATTTTAGC	ACGTTGGATGTGGAGTATTTCTCTAGCTTG	TCTCTAGCTTGCTGAAATAATG
rs522616	ACGTTGGATGCGTAGCTGCTCCATAAATAG	ACGTTGGATGACAGAGAGAATTTCAGTCCG	gaCGGTAAGCAATGTAATTCATTTCA

### Statistical analysis

We used Chi-squared test to compare the distribution of categorical variables and Student’s t-test to compare continuous variables [32]. The Hardy-Weinberg equilibrium (HWE) of each SNP was assessed in order to compare the expected frequencies of the genotypes in the control groups. The minor allele was regarded as a risk allele for OA susceptibility. Allele frequencies and genotype frequencies for each SNP of OA patients and control subjects were compared using χ^2^ test. Odds ratios (ORs) and 95% confidence intervals (CIs) were tested by unconditional logistic regression analysis to evaluate the SNPs’ effects on the risk of OA in the five models (codominant, dominant, recessive, over-dominant and log-additive). All statistical analyses were performed using SPSS version 17.0 statistical package (SPSS, Chicago, IL, USA) and Microsoft Excel (Microsoft, Redmond, WA, USA). A *p* < 0.05 was considered statistically significant and all statistical tests were two-sided. Haploview software package (version4.2) and SHEsis software platform (http://www.nhgg.org/analysis/) were used to analyze linkage disequilibrium, haplotype construction, and genetic association at polymorphism loci. Bonferroni correction was used to adjust for multiple tests.

## Abbreviations

SNP: single nucleotide polymorphism
*MMP-3*: *matrix metalloproteinase-3*
OA: osteoarthritis
MAF: minor allele frequencies
HWE: Hardy-Weinberg Equilibrium
OR: odds ratio
CI: confidence interval
