# Correction: *MMP*-3 gene polymorphisms are associated with increased risk of osteoarthritis in Chinese men

**DOI:** 10.18632/oncotarget.25223

**Published:** 2018-04-13

**Authors:** Wen Guo, Pengcheng Xu, Tianbo Jin, Jihong Wang, Dongsheng Fan, Zengtao Hao, Yuntao Ji, Shangfei Jing, Chaoqian Han, Jieli Du, Dong Jiang, Shuzheng Wen, Jianzhong Wang

**Affiliations:** ^1^ Inner Mongolia Medical University, Hohhot, Inner Mongolia, China; ^2^ National Engineering Research Center for Miniaturized Detection Systems, School of Life Sciences, Northwest University, Xi'an, Shaanxi, China; ^3^ Xi'an Tiangen Precision Medical Institute, Xi'an, Shaanxi, China; ^4^ Department of Hand and Foot Surgery, Second Affiliated Hospital, Inner Mongolia Medical University, Hohhot, Inner Mongolia Autonomous Region, China; ^5^ Department of Trauma, Second Affiliated Hospital, Inner Mongolia Medical University, Hohhot, Inner Mongolia Autonomous Region, China; ^6^ Department of Hand Surgery, Hebei province Cangzhou Hospital of integrated Traditional and Western Medicine, Cangzhou, Hebei, China; ^7^ Cangzhou People's Hospitial, Cangzhou, Hebei, China

**This article has been corrected:** The correct author affiliations are given below:

^1^ Inner Mongolia Medical University, Hohhot, Inner Mongolia, China

^2^ National Engineering Research Center for Miniaturized Detection Systems, School of Life Sciences, Northwest University, Xi'an, Shaanxi, China

^3^ Xi'an Tiangen Precision Medical Institute, Xi'an, Shaanxi, China

^4^ Department of Hand and Foot Surgery, Second Affiliated Hospital, Inner Mongolia Medical University, Hohhot, Inner Mongolia Autonomous Region, China

^5^ Department of Trauma, Second Affiliated Hospital, Inner Mongolia Medical University, Hohhot, Inner Mongolia Autonomous Region, China

^6^ Department of Hand Surgery, Hebei province Cangzhou Hospital of integrated Traditional and Western Medicine, Cangzhou, Hebei, China

^7^ Cangzhou People's Hospitial, Cangzhou, Hebei, China

^*^ These authors have contributed equally to this work

Original article: Oncotarget. 2017; 8:79491-79497. https://doi.org/10.18632/oncotarget.18493

